# Characterizing the long-term variation in grain quality of super hybrid rice under the interactive effects of nitrogen and climate

**DOI:** 10.3389/fpls.2026.1820987

**Published:** 2026-09-16

**Authors:** Xinzhen Zhang, Zhiqiang Guo, Jianwu Li, Juan Yang, Tian Liu, Qian Hu, Xiaqin Luo, Gui Xiao, Wanju Shi

**Affiliations:** 1 College of Agronomy, Hunan Agricultural University, Changsha, China; 2 State Key Laboratory of Hybrid Rice, Hunan Hybrid Rice Research Center, Changsha, China; 3 Yuelushan Laboratory, Changsha, China

**Keywords:** climate, grain qualities, nitrogen, super hybrid rice, variety

## Abstract

Super hybrid rice is widely cultivated for its high yield potential. However, systematic knowledge of how its grain quality has evolved alongside genetic yield improvement, and how it responds to variety factors, nitrogen (N) management, and climatic conditions, remains limited. Two-year field experiments were conducted at two subtropical sites using three representative super hybrid rice varieties representing different breeding phases and grown under four N application rates. In addition, historical grain quality data from all 136 super hybrid rice varieties released between 2005 and 2023 were analyzed to assess long-term trends associated with breeding progress. The historical analysis revealed that head rice rate increased by 0.25% per year, whereas chalky grain rate, chalkiness degree, and amylose content declined significantly over the same period. Consistent with these trends, field experiments showed that cultivars from more recent breeding phases exhibited higher head rice rates, lower chalkiness, and lower protein and amylose contents than earlier varieties. Random forest and structural equation modeling indicated that variety was an important contributor of variation in head rice rate, appearance traits, and amylose content under the present field conditions, with RF relative importance values ranging from 12.25% to 26.87% for these traits. Pre-anthesis temperature and solar radiation primarily influenced milling quality and chalkiness, whereas post-anthesis high temperature exerted stronger effects on grain morphology and starch-related traits. Nitrogen application showed trait-specific effects, with a strong association with protein content in the RF analysis (relative importance = 10.09%) and additional effects on milling and appearance traits under some N treatments. This study provides evidence that released super hybrid rice varieties showed clear changes in grain quality over release years, and that variety, nitrogen management and climatic conditions jointly affected grain quality traits under field conditions.

## 1 Introduction

Rice is the staple food for over half of the global population, and its sustained increase in production is paramount for food security (Sen et al., 2020). To address this need, China launched the landmark Super Hybrid Rice Breeding Program in 1996, achieving notable improvements in yield and adaptability (Cheng et al., 2007). Representative varieties developed across successive breeding phases, such as Liangyoupeijiu (LYPJ, Phase I), Y-liangyou 1 (YLY1, Phase II), and Y-liangyou 900 (YLY900, Phase IV), illustrate the continuous increase in yield over time, with modern varieties achieving approximately 12% higher yield than conventional hybrids (Huang et al., 2017a; Yuan, 2015). Despite substantial yield gains, grain quality is considered to have lagged behind in many super hybrid rice cultivars, which may constrain adoption and market value compared with traditional high-quality varieties (Xu et al., 2022). Consequently, achieving the synergistic improvement of high yield and desirable grain quality has become a central challenge in contemporary rice breeding, underscoring the need to evaluate grain quality alongside yield in super hybrid rice breeding programs.

Grain quality, encompassing complex traits such as milling, appearance, cooking, eating, and nutritional properties, has recently become a major breeding objective and a critical determinant of market value (Custodio et al., 2019). In China, sustained efforts in quality-oriented breeding have led to substantial achievements in rice grain quality over recent decades, resulting in the successful release and wide adoption of numerous high-quality indica and japonica cultivars (Zhou et al., 2020). However, analyses of variety data from previous breeding programs have shown that the patterns of grain quality improvement differ markedly among rice types, including conventional indica and japonica, indica and japonica hybrids, and indica–japonica hybrid rice (Feng et al., 2017; Yang et al., 2025a; Zeng et al., 2019). Despite these advances, the temporal variation of grain quality traits in super hybrid rice has not yet been systematically investigated. In super hybrid rice, recent molecular and marker-assisted breeding efforts have demonstrated that key grain quality genes, such as *Wx* and *ALK*, can be effectively introgressed into elite hybrid parents, highlighting the potential for genetic improvement of grain quality in super hybrid rice (Qian et al., 2016). Previous studies comparing the representative super hybrid rice cultivar Liangyoupeijiu (LYPJ) with conventional hybrid rice Shanyou 63 (Wang et al., 2012), and the elite inbred cultivar Huanghuazhan (Huang et al., 2017b) have shown that LYPJ achieved a comparable head rice yield but exhibited inferior appearance quality. Nevertheless, systematic comparisons of grain quality variation among different super hybrid rice varieties across breeding stages remain limited.

Rice grain quality is determined not only by variety, but also by crop management and environmental conditions, among which nitrogen (N) application and temperature are recognized as two of the most influential factors (Fitzgerald et al., 2009). Nitrogen regulates starch granule size and amylopectin chain structure (Tang et al., 2019; Zhou et al., 2022a), as well as storage protein composition in the endosperm (Xiong et al., 2023; Tu et al., 2025), thereby influencing rice grain quality. These findings indicate that the effects of nitrogen on grain quality are trait-specific and depend on nitrogen rate, application timing, and the target quality trait. Temperature affects photosynthetic efficiency, assimilate translocation to the developing endosperm (He et al., 2025), and the activities of starch-biosynthetic enzymes, thereby influencing starch accumulation, starch granule structure, and protein content, and ultimately determining grain quality (Tang et al., 2018). Previous studies have extensively documented the effects of nitrogen management and temperature on grain yield in super hybrid rice (Lin et al., 2009; Liu et al., 2019); however, their impacts on grain quality have received comparatively less attention. In high-yield production systems of super hybrid rice, nitrogen management is a key lever for balancing yield and quality, and super hybrid rice exhibits distinct nitrogen response patterns compared with ordinary hybrid rice (Huang et al., 2018). Therefore, elucidating the combined effects of nitrogen and temperature on grain quality is critical for developing optimized management strategies for super hybrid rice.

However, despite the well-documented yield gains of super hybrid rice, systematic understanding of how its grain quality has evolved across successive breeding phases, and how it responds to key agronomic practices (e.g., nitrogen fertilization) and climatic conditions, remains limited. To address these gaps, grain quality traits of 136 super hybrid rice varieties released between 2005 and 2023 were analyzed to characterize release-year-associated trends in grain quality. In parallel, two-year field experiments were conducted at two subtropical sites using three representative super hybrid rice varieties from different breeding phases, each grown under four nitrogen application rates. Specifically, this study aimed to: (1) determine whether and how grain quality changed over the release years of super hybrid rice varieties; and (2) quantify the relative importance of variety identity, nitrogen management, and climatic conditions in determining individual grain quality traits under the present field conditions. We hypothesized that grain quality traits of released super hybrid rice varieties have changed over release years, and that variety, nitrogen management, and climatic factors exert trait-specific contributions to grain quality under field conditions.

## 2 Materials and methods

### 2.1 Experimental site and design

Field experiments were conducted in 2021 and 2022 at Longhui (27°28′N, 110°56′E, 385 m above sea level (asl)) and Changsha (27°12′N, 113°16′E, 67 m asl) in Hunan Province, China. Prior to basal fertilizer application, soil samples were collected from the upper 0–20 cm soil layer for analysis. The average soil properties across the two experimental years indicated a clay loam texture. At Longhui, the soil had a pH of 5.83, organic matter content of 34.0 g·kg^–1^, total nitrogen of 1.8 g·kg^–1^, Olsen P of 17.8 mg·kg^–1^, and available potassium of 214.0 mg·kg^–1^. At Changsha, the corresponding values were pH 5.70, organic matter 23.3 g·kg^–1^, total nitrogen 1.4 g·kg^–1^, Olsen P 6.1 mg·kg^–1^, and available potassium 198.2 mg·kg^–1^.

The experiments were arranged in a split-plot design, with nitrogen (N) treatments as the main plots and rice varieties as the subplots, and three replications. Each experimental plot was 30 m^2^. Each plot was separated by a wide ridge lined with plastic film inserted into the soil to a depth of approximately 0.5 m to prevent N and water exchange between plots.

### 2.2 Crop management

In the Chinese Super Hybrid Rice Breeding Program, representative cultivars have been developed across successive phases of yield improvement, with Liangyoupeijiu (LYPJ) representing Phase I, Y-liangyou 1 (YLY1) representing Phase II, and Y-liangyou 900 (YLY900) representing later Phase IV achievements (Yuan, 2015). These cultivars, representing successive increases in yield targets, were selected as representative benchmark cultivars from different breeding phases of super hybrid rice. Detailed varietal information is provided in **Supplementary Table 1**. Seeds were treated to break dormancy, pre-germinated, and manually sown in seedling beds. Twenty-five days after emergence, seedlings were transplanted manually at a spacing of 0.2 m × 0.2 m with two seedlings per hill.

Nitrogen fertilizer was applied at four rates, 0 kg N ha^-1^ (N0), 150 kg N ha^-1^ (N1), 240 kg N ha^-1^ (N2) and 330 kg N ha^-1^ (N3), in both 2021 and 2022. These N application rates were selected to represent a no-nitrogen input, a low-to-medium input, the typical nitrogen application level of local farmers (Deng et al., 2022), and a high nitrogen rate used in high-yield production system in previous studies (Li et al., 2014; Xu et al., 2021). Urea was used as the N source. For each treatment, basal N fertilizer accounted for 50% of the total N and was incorporated into the soil one day before transplanting. The remaining nitrogen was split-applied at the mid-tillering (30%) and panicle initiation (20%) stages. Phosphorus (30 kg P ha^-1^ as single superphosphate) was manually applied and incorporated into all plots one day before transplanting. Potassium fertilizer (K_2_O, 150 kg ha^-1^) was applied at the basal application (50%) and panicle initiation stage (50%).

A water depth of approximately 5 cm was maintained in the fields from transplanting until one week before harvest, except for a one-week drainage period at maximum tillering to reduce unproductive tillers. Weeds, insects and diseases were effectively controlled using chemical measures as required. Photosynthetically active radiation and temperature data were obtained from an automatic weather station (EM50, Decagon Devices, USA) located adjacent to the experimental sites.

### 2.3 Sampling and measurements

At maturity, grain samples were harvested from a 5 m^2^ central area in each plot, excluding border rows and off-type plants. Filled grains were air-dried and stored at room temperature for three months before grain quality trait assessment. Brown rice (%) was recorded after dehulling. Milled rice was obtained by polishing the brown rice, and head rice was measured as the weight of unbroken milled grains relative to the total sample weight. Three replicates of at least 200 milled rice grains each were then scanned using a flatbed scanner (ScanMaker i800 Plus, Microtek, Shanghai, China) and analyzed with SC-E software (Hangzhou, Wanshen Detection Technology Co., Ltd., Hangzhou, China) to determine grain length, grain width, grain aspect ratio, chalkiness degree (defined as the percentage of all chalky area relative to the total projected grain area), and chalky grain rate (defined as the percentage of chalky grains).

Amylose content was measured according to the national standard of the People’s Republic of China GB/T 17891-2017, based on the iodine colorimetric method. Briefly, 100 mg of rice flour was digested in 1 mL of 95% ethanol and 9 mL of 1 M NaOH, boiled for 10 min, cooled, and diluted to 100 mL. An aliquot of 5 mL was mixed with 1 mL of 1 M acetic acid and 2 mL of iodine solution (0.2% I_2_ + 2% KI), then made up to 100 mL. The absorbance was measured at 620 nm. Protein content was indirectly measured using the semi-micro Kjeldahl method to estimate the nitrogen concentration, and a Kjeldahl conversion coefficient of 5.95 was applied, referring to the national standards of the People’s Republic of China (GB/T 5009.5-2016).

### 2.4 Variety dataset collection

Historical grain quality data for all 136 nationally or provincially certified indica super rice varieties released between 2005 and 2023 were obtained from the China Rice Data Centre (http://www.ricedata.cn/variety/). The dataset included brown rice rate, milled rice rate, head rice rate, grain length, grain aspect ratio, chalky grain rate, chalkiness degree, amylose content, and protein content. Variety names, release years, trait names, and measurement units were checked for consistency before analysis. For varieties certified multiple times, average values were used. No interpolation was performed for grain quality traits.

### 2.5 Statistical analysis

The effects of rice varieties, nitrogen treatments, experimental sites, and their interactions on grain quality were statistically analyzed by analysis of variance (ANOVA) using the R language (version 4.1.0; http://www.r-project.org). The split-plot design was considered, with nitrogen treatments as the main plots and varieties as the subplots. The ANOVA results are provided in **Supplementary Table 2**. Mean comparisons were performed using the least significant difference (LSD) test at *P < 0.05*. Data in all tables and figures are presented as means ± standard error.

Random forest (RF) analysis was conducted in Python (version 3.6) to quantify the relative importance of variety, nitrogen treatments, and meteorological factors in determining individual rice grain quality traits. Treatment means across three replications were used for RF analysis. The RF model served as a non-parametric ensemble approach to identify key predictors and rank their contributions without assuming linear relationships among variables. Model performance was evaluated using out-of-bag prediction, and model parameters and out-of-bag errors were provided in **Supplementary Table 3**. To further examine the relationships among these factors, a partial least squares structural equation model (PLS-SEM) was subsequently applied using SmartPLS (version 4; SmartPLS GmbH). The PLS-SEM analysis was used to characterize the associations among variety, nitrogen fertilization, meteorological conditions, and grain quality traits, thereby complementing the RF-based importance ranking. For the PLS-SEM analysis, variety was not represented by release year. Because the three varieties were selected as representative benchmark varieties from different breeding phases, a planned breeding-phase contrast was used, with LYPJ, YLY1 and YLY900 coded as -1, 0 and 1, respectively. This coding was used to examine the directional difference among the three representative cultivars in the field experiment, rather than to represent genetic background as a continuous variable.

Linear regression analysis was conducted using SAS version 9.4M7 (SAS Institute Inc., Cary, NC, USA) to examine trends in grain quality traits over the year of release for the 136 historical varieties. The model used was:

Y_i_ = α + βX_i_ + ϵi.

where Y_i_ is the grain quality trait, X_i_ is the release year of the variety i, α is the intercept, β is the slope, and ϵi is the residual error for the i-th observation.

## 3 Results

### 3.1 Climate conditions

In this study, the pre-anthesis period referred to the period from transplanting to anthesis, and the post-anthesis period referred to the period from anthesis to maturity. Throughout the entire growth period, the average daily, maximum and minimum temperatures in Longhui over the two years (2021 and 2022) were 25.5 °C, 31.3 °C, and 21.4 °C, respectively. In contrast, the corresponding values in Changsha were 28.4 °C, 33.4 °C, and 24.5 °C, indicating consistently higher temperatures in Changsha compared to Longhui (**Figure 1**). During the pre-anthesis period, temperatures in Longhui were 25.2 °C, 30.2 °C, and 21.5 °C, while in Changsha they reached 29.2 °C, 33.6 °C, and 25.7 °C. During the post-anthesis period, daily temperatures in Longhui increased to 26.2 °C, 33.3 °C, and 21.2 °C, compared with 27.2 °C, 33.1 °C, and 22.6 °C in Changsha, indicating that temperature differences were more pronounced before anthesis.

**Figure 1:**
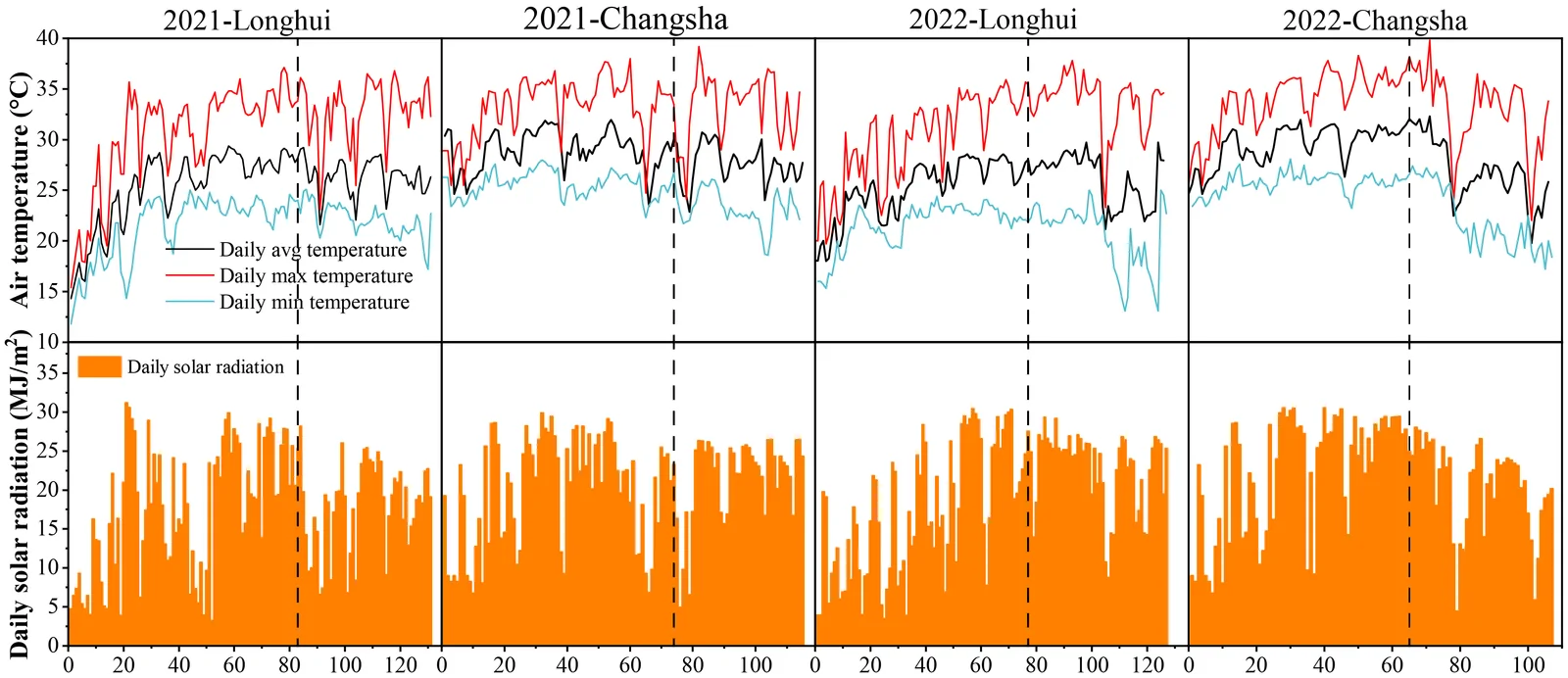
Daily air temperature, including average, maximum and minimum temperature, and daily solar radiation starting from days after transplanting across Longhui and Changsha regions during 2021 and 2022. Black dashed lines indicate the day of anthesis.

Average daily solar radiation across the season was 18.69 MJ/m^2^ in Longhui and 21.19 MJ/m^2^ in Changsha. During the pre-anthesis period, it was 17.69 MJ/m^2^ in Longhui and 21.29 MJ/m^2^ in Changsha, increasing after anthesis to 20.39 MJ/m^2^ and 21.08 MJ/m^2^, respectively, indicating consistently higher radiation in Changsha, particularly before anthesis.

At Longhui, the year-to-year climatic difference was mainly observed during the post-anthesis period. During the pre-anthesis period, the daily average, maximum and minimum temperatures were 25.23, 30.38 and 21.35 °C in 2021, respectively, compared with 25.08, 29.92 and 21.58 °C in 2022. During the post-anthesis period, the corresponding values were 26.31, 32.88 and 22.03 °C in 2021 and 26.18, 33.74 and 20.44 °C in 2022. Thus, although the post-anthesis daily average temperature was similar between years, Longhui in 2022 showed a higher post-anthesis daily maximum temperature and a larger daily temperature range than in 2021. Post-anthesis daily solar radiation at Longhui also increased from 17.98 MJ/m^2^ in 2021 to 22.81 MJ/m^2^ in 2022.

### 3.2 Milling qualities

ANOVA showed that site, variety, and nitrogen treatment significantly affected most grain quality traits in both years, with several significant two-way and three-way interactions (**Supplementary Table 2**). Across both sites and years, the brown rice rate and milled rice rate of LYPJ, YLY1, and YLY900 were significantly lower under the zero N treatment (N0) than under all N-fertilized treatments (N1- N3), with the exception of the milled rice rate of YLY1 at Longhui and LYPJ at Changsha in 2022 (**Figure 2**). Once N was applied, increasing N rates from 150 to 330 kg N ha^–1^ generally resulted in only minor or non-significant changes in brown rice rate and milled rice rate across sites, years, and varieties. In Longhui, no significant differences among N1, N2, and N3 were observed for either trait in 2021, and a similar pattern was found for the milled rice rate in 2022. Although the brown rice rate under N1 was lower than under N2 and N3 in 2022, the overall response to increasing N beyond 150 kg N ha^–1^ remained limited. In Changsha, both brown rice rate and milled rice rate increased significantly from N1 to N2 in some cases; however, no consistent or systematic differences were detected between N2 and N3, except for the brown rice rate of LYPJ and the milled rice rate of LYPJ and YLY1 in 2021.

**Figure 2:**
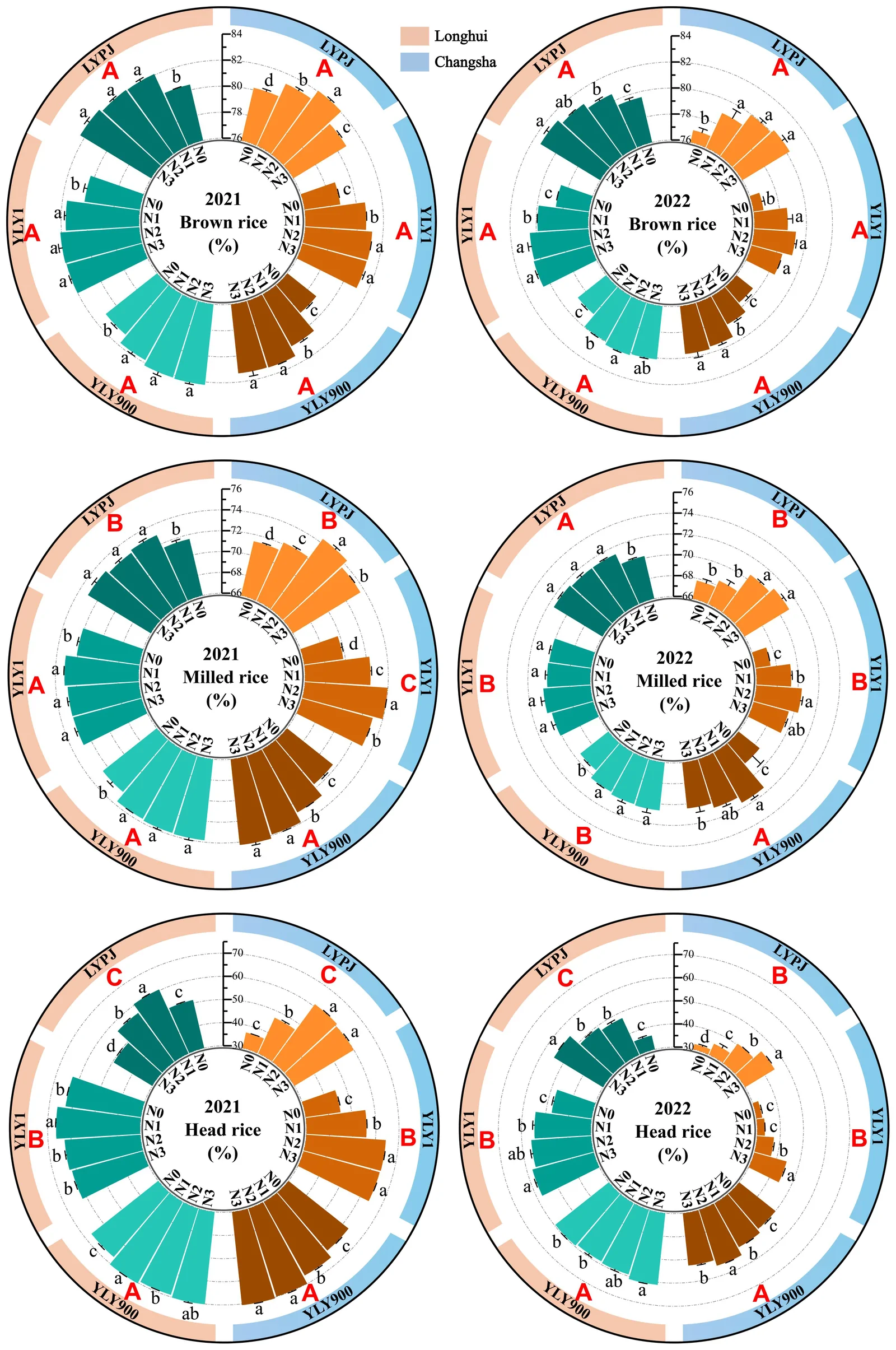
Brown rice, milled rice and head rice percentages of three super hybrid rice varieties (LYPJ, YLY1 and YLY900) grown under four nitrogen treatments (N0, N1, N2 and N3) in Longhui and Changsha regions during 2021 and 2022. Bars represent mean ± standard error. Different lowercase letters indicate significant differences among nitrogen treatments within the same variety (*p < 0.05*), and different uppercase letters indicate significant differences among varieties (*p < 0.05*).

For head rice rate, the lowest values were generally observed under the zero N treatment (N0) for all three varieties across both sites and years. In Longhui in 2021, the head rice rate reached its maximum at the N1 level for all three varieties, whereas in Changsha in 2021 and across both sites in 2022, the highest head rice rate was observed at N2 and/or N3. Across varieties, the head rice rate increased significantly from LYPJ to YLY900, with YLY900 exhibiting the highest values.

### 3.3 Appearance qualities

In most cases, grain length, grain width, and grain aspect ratio of the three varieties were higher under nitrogen application (N1–N3) than under the zero-nitrogen treatment (N0), except for grain length and grain aspect ratio in Longhui in 2021 (**Tables 1**, **2**). The highest chalky grain rate and chalkiness degree for three rice varieties were observed at N0 levels across two sites and years, except for YLY1 in Longhui in two years. In contrast, the lowest chalky grain rate and chalkiness degree were observed at N3 for all three varieties in Changsha across both years. In Longhui, however, the lowest values occurred at N3 for YLY900 and at N1 for YLY1 in both years.

**Table 1:** Appearance qualities of super hybrid rice varieties LYPJ, YLY1 and YLY900 under four nitrogen treatments (N0-N3) at Longhui and Changsha in 2021.

Site	Variety	Nitrogen	Grain length (mm)	Grain width (mm)	Grain aspect	Chalky grain rate (%)	Chalkiness degree (%)
					ratio		
Longhui	LYPJ	N0	6.484 ± 0.011 a	2.217 ± 0.003 c	2.93 ± 0.01 a	34.2 ± 0.6 a	11.0 ± 0.2 a
		N1	6.411 ± 0.011 b	2.236 ± 0.004 a	2.87 ± 0.01 b	32.2 ± 0.7 b	9.4 ± 0.3 c
		N2	6.328 ± 0.006 c	2.219 ± 0.002 bc	2.86 ± 0.01 b	35.5 ± 0.4 a	10.6 ± 0.2 ab
		N3	6.300 ± 0.001 c	2.229 ± 0.004 ab	2.83 ± 0.01 c	35.2 ± 0.5 a	10.2 ± 0.1 b
		**Mean**	**6.381 ± 0.042 B**	**2.225 ± 0.004 B**	**2.87 ± 0.01 B**	**34.3 ± 0.5 A**	**10.3 ± 0.2 A**
	YLY1	N0	6.637 ± 0.003 a	2.190 ± 0.003 b	3.04 ± 0.00 a	27.6 ± 0.2 b	8.5 ± 0.1 bc
		N1	6.618 ± 0.017 a	2.216 ± 0.003 a	3.00 ± 0.00 b	26.2 ± 0.4 b	8.0 ± 0.2 c
		N2	6.497 ± 0.010 b	2.211 ± 0.004 a	2.94 ± 0.00 d	31.8 ± 0.3 a	10.1 ± 0.4 a
		N3	6.514 ± 0.002 b	2.204 ± 0.004 a	2.96 ± 0.00 c	32.2 ± 0.7 a	9.3 ± 0.2 ab
		**Mean**	**6.567 ± 0.036 A**	**2.205 ± 0.006 C**	**2.99 ± 0.01 A**	**29.5 ± 0.8 B**	**9.0 ± 0.3 B**
	YLY900	N0	6.314 ± 0.007 a	2.212 ± 0.004 b	2.86 ± 0.00 a	14.3 ± 0.2 a	4.3 ± 0.1 a
		N1	6.236 ± 0.006 b	2.243 ± 0.004 a	2.78 ± 0.00 b	10.9 ± 0.4 b	3.4 ± 0.2 b
		N2	6.219 ± 0.010 b	2.236 ± 0.001 a	2.79 ± 0.00 b	11.5 ± 0.3 b	3.1 ± 0.1 b
		N3	6.184 ± 0.004 c	2.243 ± 0.001 a	2.76 ± 0.00 c	11.4 ± 0.6 b	3.2 ± 0.2 b
		**Mean**	**6.238 ± 0.027 C**	**2.233 ± 0.007 A**	**2.80 ± 0.01 C**	**12.0 ± 0.4 C**	**3.5 ± 0.2 C**
Changsha	LYPJ	N0	6.382 ± 0.019 c	2.093 ± 0.009 b	3.06 ± 0.00 c	57.5 ± 1.2 a	22.0 ± 0.9 a
		N1	6.552 ± 0.012 b	2.121 ± 0.004 a	3.10 ± 0.00 b	40.6 ± 0.5 b	12.7 ± 0.2 b
		N2	6.601 ± 0.003 a	2.120 ± 0.006 a	3.12 ± 0.01 a	37.0 ± 1.3 c	10.7 ± 0.2 c
		N3	6.568 ± 0.003 ab	2.125 ± 0.000 a	3.10 ± 0.00 b	37.1 ± 0.6 c	10.5 ± 0.1 c
		**Mean**	**6.526 ± 0.049 B**	**2.115 ± 0.007 A**	**3.09 ± 0.01 B**	**43.1 ± 2.6 A**	**14.0 ± 1.4 A**
	YLY1	N0	6.420 ± 0.012 d	2.012 ± 0.002 c	3.20 ± 0.01 c	29.7 ± 0.4 a	10.9 ± 0.2 a
		N1	6.675 ± 0.003 b	2.037 ± 0.004 b	3.29 ± 0.01 a	27.9 ± 0.3 b	9.4 ± 0.2 b
		N2	6.699 ± 0.002 a	2.074 ± 0.003 a	3.24 ± 0.01 b	27.2 ± 0.1 b	8.5 ± 0.1 c
		N3	6.584 ± 0.006 c	2.067 ± 0.004 a	3.19 ± 0.01 c	24.6 ± 0.4 c	7.3 ± 0.1 d
		**Mean**	**6.595 ± 0.063 A**	**2.048 ± 0.014 C**	**3.23 ± 0.01 A**	**27.4 ± 0.6 B**	**9.0 ± 0.4 B**
	YLY900	N0	6.177 ± 0.021 c	2.068 ± 0.005 c	2.99 ± 0.00 b	25.5 ± 0.6 a	9.2 ± 0.2 a
		N1	6.298 ± 0.010 a	2.080 ± 0.003 c	3.03 ± 0.01 a	14.6 ± 0.2 b	4.8 ± 0.1 b
		N2	6.329 ± 0.012 a	2.095 ± 0.005 b	3.03 ± 0.01 a	13.7 ± 0.2 bc	4.3 ± 0.3 bc
		N3	6.230 ± 0.016 b	2.110 ± 0.001 a	2.96 ± 0.01 c	12.5 ± 0.7 c	3.7 ± 0.1 c
		**Mean**	**6.259 ± 0.034 C**	**2.088 ± 0.009 B**	**3.00 ± 0.01 C**	**16.6 ± 1.6 C**	**5.5 ± 0.7 C**

Values are means ± SE. Bold values indicate means across the four N treatments for each variety. Within each site and variety, lowercase letters indicate significant differences among N treatments; within each site, uppercase letters following the bold means indicate significant differences among varieties (P < 0.05).

**Table 2:** Appearance qualities of super hybrid rice varieties LYPJ, YLY1 and YLY900 under four nitrogen treatments at Longhui and Changsha in 2022.

Site	Variety	Nitrogen	Grain length	Grain width	Grain aspect	Chalky grain rate (%)	Chalkiness degree (%)
			(mm)	(mm)	ratio		
Longhui	LYPJ	N0	6.315 ± 0.015 c	2.182 ± 0.005 b	2.91 ± 0.00 b	52.3 ± 0.8 a	19.0 ± 0.9 a
		N1	6.366 ± 0.011 ab	2.203 ± 0.004 a	2.91 ± 0.00 b	49.4 ± 0.4 b	15.8 ± 0.4 b
		N2	6.392 ± 0.013 a	2.179 ± 0.016 b	2.94 ± 0.00 a	46.1 ± 0.4 c	13.9 ± 0.5 c
		N3	6.330 ± 0.005 bc	2.184 ± 0.005 b	2.91 ± 0.00 b	44.8 ± 0.2 c	13.1 ± 0.3 c
		**Mean**	**6.351 ± 0.017 B**	**2.187 ± 0.005 A**	**2.92 ± 0.01 B**	**48.2 ± 0.9 A**	**15.5 ± 0.7 A**
	YLY1	N0	6.501 ± 0.013 b	2.159 ± 0.006 a	3.03 ± 0.00 a	37.0 ± 1.0 b	11.3 ± 0.6 a
		N1	6.537 ± 0.001 a	2.163 ± 0.003 a	3.03 ± 0.00 a	34.7 ± 1.0 c	9.7 ± 0.4 b
		N2	6.507 ± 0.004 b	2.159 ± 0.010 a	3.03 ± 0.01 a	34.9 ± 0.0 c	10.7 ± 0.0 ab
		N3	6.490 ± 0.004 b	2.151 ± 0.004 a	3.03 ± 0.00 a	39.3 ± 0.5 a	11.3 ± 0.1 a
		**Mean**	**6.509 ± 0.010 A**	**2.158 ± 0.003 B**	**3.03 ± 0.00 A**	**36.5 ± 0.6 B**	**10.8 ± 0.3 B**
	YLY900	N0	6.125 ± 0.011 b	2.146 ± 0.008 a	2.86 ± 0.01 bc	30.8 ± 0.4 a	10.5 ± 0.3 a
		N1	6.105 ± 0.008 b	2.151 ± 0.004 a	2.85 ± 0.00 c	30.6 ± 0.6 a	10.3 ± 0.3 ab
		N2	6.179 ± 0.002 a	2.139 ± 0.006 a	2.90 ± 0.01 a	28.6 ± 0.1 b	9.9 ± 0.1 ab
		N3	6.105 ± 0.004 b	2.136 ± 0.002 a	2.87 ± 0.00 b	28.3 ± 0.1 b	9.8 ± 0.2 b
		**Mean**	**6.128 ± 0.017 C**	**2.143 ± 0.003 C**	**2.87 ± 0.01 C**	**29.6 ± 0.4 C**	**10.1 ± 0.1 B**
Changsha	LYPJ	N0	6.074 ± 0.015 d	2.030 ± 0.012 b	3.01 ± 0.01 c	53.7 ± 0.2 a	22.2 ± 0.2 a
		N1	6.355 ± 0.008 c	2.105 ± 0.007 a	3.03 ± 0.00 b	48.3 ± 0.6 b	17.0 ± 0.1 b
		N2	6.466 ± 0.009 b	2.120 ± 0.005 a	3.06 ± 0.00 a	39.6 ± 0.1 c	11.5 ± 0.2 c
		N3	6.524 ± 0.004 a	2.130 ± 0.005 a	3.08 ± 0.01 a	38.7 ± 0.1 c	11.2 ± 0.1 c
		**Mean**	**6.355 ± 0.100 B**	**2.096 ± 0.023 A**	**3.05 ± 0.01 B**	**45.1 ± 1.9 A**	**15.5 ± 1.4 A**
	YLY1	N0	6.168 ± 0.010 c	2.004 ± 0.001 c	3.10 ± 0.00 b	49.6 ± 0.2 a	18.3 ± 0.1 a
		N1	6.504 ± 0.017 b	2.081 ± 0.005 b	3.15 ± 0.01 a	41.8 ± 0.7 b	12.9 ± 0.7 b
		N2	6.573 ± 0.006 a	2.104 ± 0.009 a	3.15 ± 0.01 a	34.8 ± 0.4 c	9.5 ± 0.3 c
		N3	6.537 ± 0.005 b	2.099 ± 0.008 ab	3.13 ± 0.01 a	31.5 ± 0.2 d	9.0 ± 0.1 c
		**Mean**	**6.446 ± 0.094 A**	**2.072 ± 0.023 B**	**3.13 ± 0.01 A**	**39.4 ± 2.1 B**	**12.4 ± 1.1 B**
	YLY900	N0	5.889 ± 0.021 c	2.019 ± 0.012 c	2.93 ± 0.01 c	27.5 ± 0.4 a	9.4 ± 0.2 a
		N1	6.058 ± 0.009 b	2.060 ± 0.005 b	2.96 ± 0.00 b	21.4 ± 0.2 b	6.5 ± 0.2 b
		N2	6.326 ± 0.014 a	2.103 ± 0.005 a	3.02 ± 0.00 a	16.9 ± 0.5 d	4.5 ± 0.1 d
		N3	6.262 ± 0.038 a	2.097 ± 0.008 a	3.00 ± 0.01 a	18.4 ± 0.2 c	5.6 ± 0.1 c
		**Mean**	**6.134 ± 0.100 C**	**2.070 ± 0.019 B**	**2.98 ± 0.01 C**	**21.1 ± 1.2 C**	**6.5 ± 0.6 C**

Values are means ± SE. Bold values indicate means across the four N treatments for each variety. Within each site and variety, lowercase letters indicate significant differences among N treatments; within each site, uppercase letters following the bold means indicate significant differences among varieties (P < 0.05).

Significant varietal differences in appearance quality were detected across both experimental sites and years. Grain length and grain aspect ratio consistently followed the order YLY1 > LYPJ > YLY900, whereas grain width varied substantially among sites and years. LYPJ generally exhibited the greatest grain width, except in Longhui in 2021, while differences between YLY1 and YLY900 were inconsistent across years. Chalky grain rate and chalkiness degree also exhibited a consistent ranking of LYPJ > YLY1 > YLY900 across sites and years, indicating the lowest chalkiness in YLY900. However, YLY900 still showed a marked year-to-year increase in chalky grain rate and chalkiness degree at Longhui in 2022 under N1–N3 compared with the corresponding treatments in 2021.

### 3.4 Nutritional quality

Protein content of the three rice varieties was lowest under the N0 levels at both sites across the two years and increased with nitrogen application, generally reaching the highest values at N3 (**Figure 3**). In 2021, amylose content showed no significant differences among nitrogen treatments for LYPJ in Longhui or for YLY1 at either site. By contrast, the highest amylose content was observed at N0 for LYPJ in Changsha and for YLY900 at both sites. In 2022, amylose content showed inconsistent responses to nitrogen application among varieties and sites.

**Figure 3:**
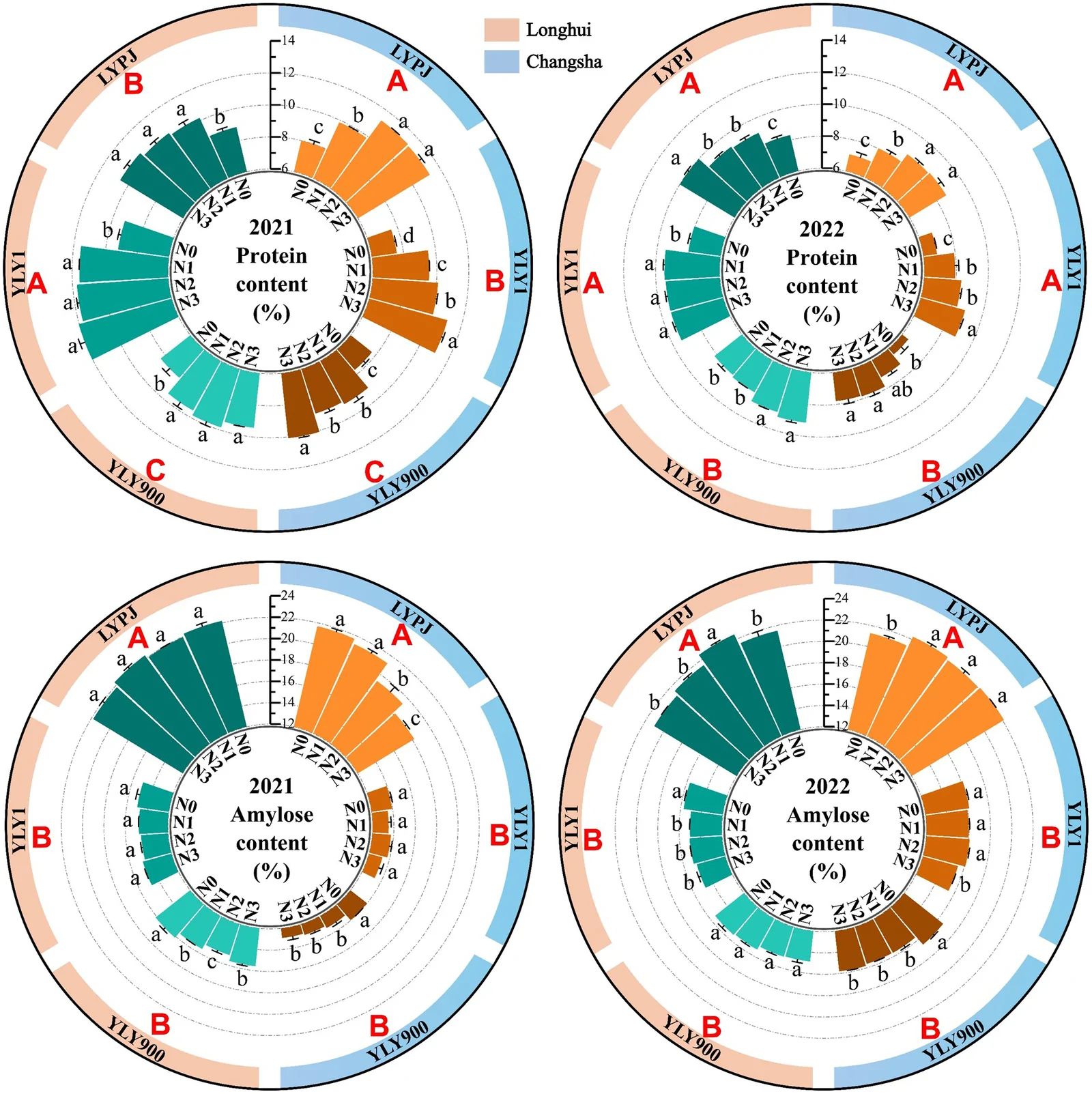
Protein and amylose content of three super hybrid rice varieties (LYPJ, YLY1 and YLY900) grown under different levels of nitrogen treatment (N0, N1, N2 and N3) across Longhui and Changsha regions during 2021 and 2022. Bars represent mean ± standard error. Different lowercase letters indicate significant differences among nitrogen treatments within the same variety (*p < 0.05*), and different uppercase letters indicate significant differences among varieties (*p < 0.05*).

Among the three varieties, YLY900 consistently exhibited the lowest protein content across both sites and years. Significant differences in protein content were observed between LYPJ and YLY1 at both sites in 2021, whereas no significant differences were observed in 2022. For amylose content, LYPJ consistently showed the highest values across sites and years, whereas YLY1 and YLY900 were comparable and both significantly lower than LYPJ.

### 3.5 Changes in grain quality of super hybrid rice varieties over the release years

From 2005 to 2023, the brown rice rate and milled rice rate of released super hybrid rice varieties showed slight increasing trends over time; however, these trends were not statistically significant (*P > 0.05*, **Figure 4**). In contrast, the head rice rate increased significantly at an average rate of 0.25 percentage points per year (*P < 0.05*), indicating a clear improvement in head rice rate over the release period. Grain length exhibited a weak, non-significant increasing trend over time (*P > 0.05*), whereas grain aspect ratio increased significantly over the years (*P < 0.01*), reflecting a gradual shift toward more slender grain shape among released varieties. Chalky grain rate and chalkiness degree both decreased markedly over time, declining by 1.88 and 0.49 percentage points per year, respectively (*P < 0.01*), indicating substantial improvement in grain appearance quality. Amylose content showed a significant downward trend, with an average annual decrease of 0.34 percentage points, whereas protein content exhibited no significant temporal trend (*P > 0.05*).

**Figure 4:**
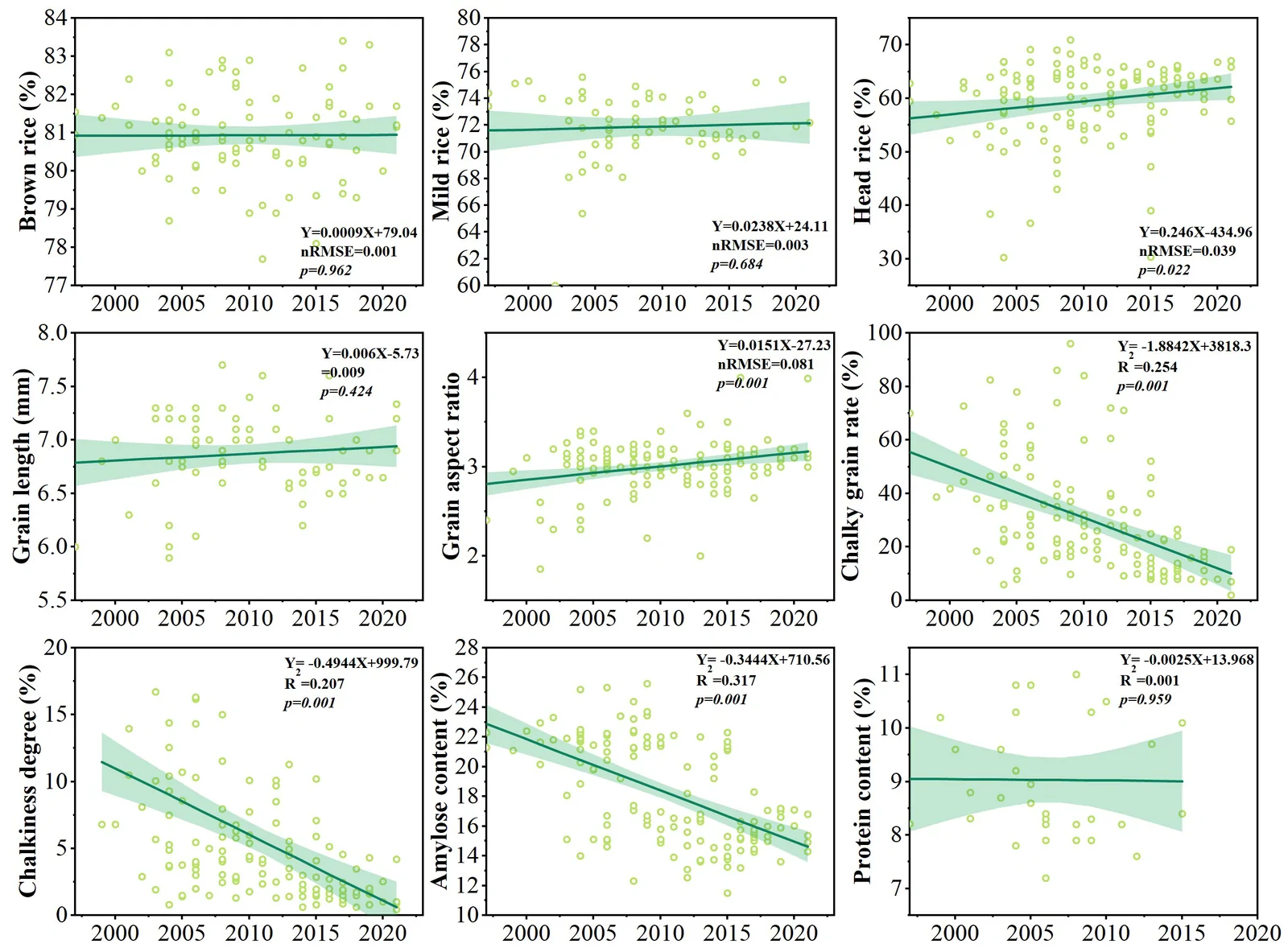
Temporal trends in grain quality traits of released super hybrid rice varieties from 2005 to 2023. Each point represents individual variety, solid lines indicate fitted regression lines, and shaded areas denote 95% confidence bands. The linear regression equations, normalized root mean square error (nRMSE), and significance levels (*p*) are provided.

### 3.6 Impact of variety, nitrogen, and weather on grain quality

Variety, nitrogen fertilizer, and climatic factors were used as independent variables in random forest models to quantify their relative importance in determining individual grain quality traits (**Figure 5**). The model parameters and out-of-bag validation results are provided in **Supplementary Table 3**. Variety was the most important factor for head rice, grain length, grain aspect ratio, chalky grain rate, chalkiness degree, and amylose content, with relative importance values ranging from 12.25% to 26.87%. Meteorological variables before and after anthesis consistently ranked among the top contributors to grain quality variation. Among climatic variables, pre-anthesis thermal conditions and post-anthesis maximum temperature were particularly influential. Compared with varietal and meteorological factors, nitrogen fertilizer generally showed lower importance for most grain quality traits. However, nitrogen fertilizer showed a clear contribution to protein content, for which it ranked among the top three predictors, with a relative importance value of 10.09%.

**Figure 5:**
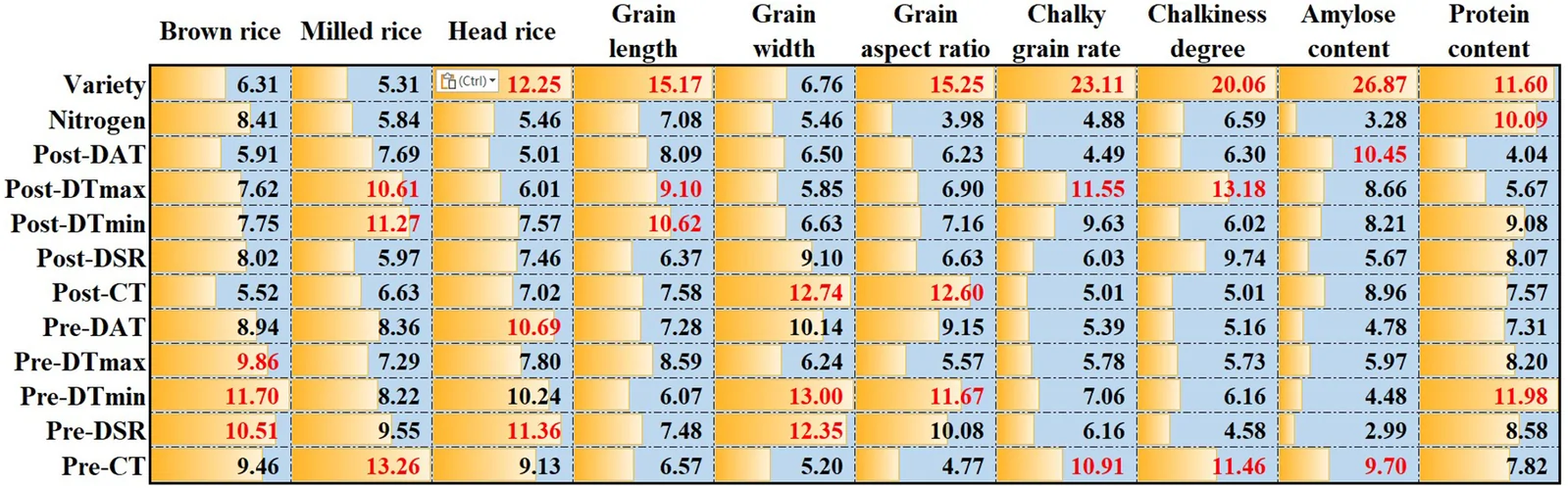
Relative importance of variety, nitrogen fertilizer and meteorological factors on grain quality predicted by the random forest model. Values were normalized to the sum of all predictors for each grain quality trait. Pre-DAT: pre-anthesis daily temperature; Pre-DTmax: pre-anthesis daily maximum temperature; Pre-DTmin: pre-anthesis daily minimum temperature; Pre-CT: pre-anthesis cumulative temperature; Pre-DSR: pre-anthesis daily solar radiation; Post-DAT: post-anthesis daily temperature; Post-DTmax: post-anthesis daily maximum temperature; Post-DTmin: post-anthesis daily minimum temperature; Post-CT: post-anthesis cumulative temperature; Post-DSR: post-anthesis daily solar radiation. Values represent percentage of relative importance. Red numbers indicate the top three most important factors for each grain quality trait.

Building on these findings, the PLS-SEM analysis further showed the relationships among these factors and grain quality traits (**Figure 6**). The breeding-phase contrast showed relationships with multiple grain quality components, including milling quality, appearance traits, grain morphology and amylose content. Nitrogen application showed a significant positive relationship with protein content, whereas its relationships with milling and appearance traits were limited. Pre-anthesis cumulative temperature and minimum temperature were negatively associated with chalkiness formation, while pre-anthesis solar radiation was positively associated with milling performance. In contrast, post-anthesis maximum temperature was associated with grain morphology, showing negative associations with grain aspect ratio and grain width but a positive association with grain length. Post-anthesis temperature also showed a negative association with amylose content.

**Figure 6:**
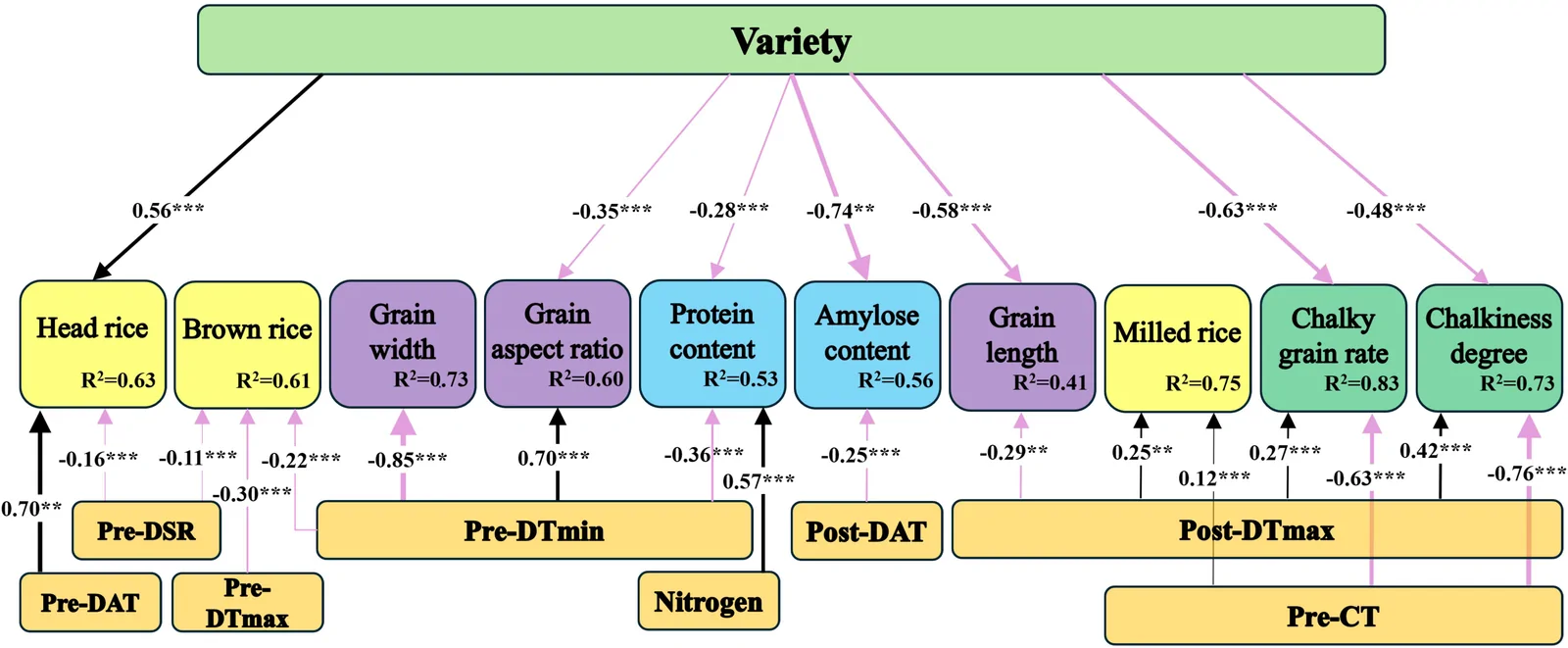
Structural equation model illustrating the associations among variety, nitrogen application, climatic factors and rice grain quality traits. Path model derived from partial least squares structural equation modeling (PLS-SEM). Variety was represented by a planned breeding-phase contrast in the PLS-SEM analysis, with LYPJ, YLY1 and YLY900 coded as -1, 0 and 1, respectively. Standardized path coefficients are shown, with black and pink arrows indicating positive and negative associations, respectively, and arrow thickness proportional to coefficient magnitude. Numbers adjacent to arrows denote path coefficients, with significance at *p < 0.05*, *p < 0.01*, and *p < 0.001*. The coefficient of determination (R^2^) values for endogenous variables are shown inside boxes, indicating the proportion of variance explained by the model. Pre-anthesis climatic variables include daily solar radiation (Pre-DSR), cumulative temperature (Pre-CT), daily minimum temperature (Pre-DTmin), daily maximum temperature (Pre-DTmax), and daily average temperature (Pre-DAT), whereas post-anthesis variables include daily maximum temperature (Post-DTmax) and daily average temperature (Post-DAT).

## 4 Discussion

### 4.1 Genetic improvement of grain quality in super hybrid rice

In recent years, with rising living standards and advances in rice breeding, the goals of rice production have shifted from solely increasing yield to improving quality while maintaining high yields. Head rice rate is a key indicator of milling quality and market value, as a higher proportion of intact grains directly increases farmers’ economic returns (Buggenhout et al., 2013). Our field experiments, combined with analyses of historical data for released super hybrid rice varieties, showed that head rice rate has steadily increased over release years. This trend is consistent with nationwide improvements in head rice rate observed for most rice types in China, except for indica–japonica hybrids (Lu et al., 2024), and with reported trends in southern indica hybrid rice (Feng et al., 2017). Released-variety datasets have been widely used to evaluate long-term changes in yield and quality traits in rice breeding studies (Feng et al., 2017; Zeng et al., 2019; Li et al., 2019; Lu et al., 2024; Yang et al., 2025a). Such official variety records do not represent a single common-environment trial, but they reflect the certified performance of released varieties in their target production regions and are suitable for characterizing historical trends among approved cultivars.

The improvement in head rice rate of super hybrid rice is particularly meaningful because milling quality is highly sensitive to environmental conditions. In Guangdong Province, the limited improvement in head rice rate over recent decades has been attributed partly to persistent high-temperature stress and a historical underemphasis on milling quality in breeding programs (Zhou et al., 2022b). Declines or no change in head rice rate have also been reported for indica inbred and indica hybrid rice under hot and humid conditions in southern China and at the national scale (Feng et al., 2017; Yang et al., 2025a; Zhou et al., 2022b). These contrasting patterns suggest that changes in head rice rate depend on both rice type and regional climatic conditions, reflecting the interaction between breeding targets and environmental constraints (Zeng et al., 2019). In the present study, the field experiment further showed that varieties from later breeding phases had higher head rice rates when grown under the same site-year and management conditions. Therefore, the increasing trend in head rice rate over release years is consistent with the contribution of varietal improvement rather than being explained only by environmental variation.

In contrast to head rice rate, both our results for super hybrid rice and previous studies on other rice types show that chalkiness and amylose content have steadily declined over release years (Zeng et al., 2019; Zhou et al., 2022b; Yang et al., 2025a; Lu et al., 2024). At the genetic level, changes in starch-related quality traits may be partly associated with the selection and introgression of major quality genes. For example, Wx is closely related to amylose synthesis, whereas ALK is associated with starch physicochemical properties such as gelatinization temperature. The use of favorable alleles at these loci in elite hybrid parents provides a genetic basis for improving eating and cooking quality in super hybrid rice (Qian et al., 2016; Zhou et al., 2020). High temperature during grain filling generally increases chalkiness and deteriorates appearance quality by affecting endosperm development, starch biosynthesis, and grain filling processes (Lin et al., 2010; Chen et al., 2017; Tang et al., 2018; Zhang et al., 2021). Therefore, the observed reduction in chalkiness over release years is unlikely to be explained simply by favorable climatic shifts. Instead, it is consistent with sustained selection for improved appearance quality in modern super hybrid rice breeding. The lower chalkiness observed in YLY900 than in LYPJ and YLY1 under the same field environments further supports this interpretation.

By comparison, grain protein content did not exhibit systematic changes over the breeding timeline in this study, consistent with findings from other rice types and regions (Zhou et al., 2022b; Lu et al., 2024). This is likely because historical breeding objectives primarily focused on appearance and favorable eating quality, as elevated protein content is generally associated with reduced rice softness and stickiness after cooking (Feng et al., 2017; Yang et al., 2025b). Consequently, protein content has not been a direct target in mainstream quality-oriented breeding programs. Overall, our results indicate that released super hybrid rice varieties have shown clear improvement in several key quality traits, including higher head rice rate, reduced chalkiness, and lower amylose content, while maintaining the high-yield potential that defines super hybrid rice.

### 4.2 Integrated effects of variety, nitrogen management, and climate on grain quality of super hybrid rice

Compared with previous studies that mainly focused on the independent effects of nitrogen on grain quality in super hybrid rice (Huang et al., 2017b; Wang et al., 2012), the present study provides an integrated assessment of the relative contributions of variety identity, nitrogen application and climatic factors to variation in grain quality of super hybrid rice. Using multi-site field data collected over two years and applying random forest models together with PLS-SEM, variety identity was an important source of variation in milling quality, appearance traits, and amylose content in super hybrid rice. Although grain quality traits respond to environmental conditions (Chen et al., 2012), the present findings show that grain quality of super hybrid rice is largely determined by varieties. These findings are consistent with previous studies highlighting the central role of genetic control in grain quality enhancement (Fitzgerald et al., 2009) and support varietal selection as a key strategy for improving grain quality in super hybrid rice (Qian et al., 2016).

Beyond varietal effects, our results reveal a clear regulation of grain quality by climatic factors, a phenomenon that has received limited quantitative characterization in super hybrid rice. Specifically, pre-anthesis temperature and solar radiation mainly influenced milling quality and chalkiness, whereas post-anthesis high temperature affected grain morphology and starch-related traits more strongly. These results agree with studies in high-quality hybrid rice, where elevated temperatures during the booting stage significantly affected milling and appearance quality (Zhang et al., 2023). They also align with reports showing that minimum temperature and solar radiation during pre- and post-anthesis periods strongly influenced milling quality and amylose content in hybrid rice grown in India (Talla et al., 2025). The strong influence of pre-anthesis climate on grain quality is likely due to its effect on photosynthetic capacity and assimilate supply, which establish the source–sink balance before anthesis and affect subsequent grain growth (Tang et al., 2023; Zhen et al., 2020). In contrast, grain-filling temperature mainly affects starch and protein biosynthesis through enzyme activity, gene expression, and assimilate partitioning, which in turn alter amylose and amylopectin structure, storage protein composition, and final grain quality traits, especially chalkiness (Lin et al., 2010; Zhang et al., 2021). While previous studies have largely focused on the role of grain-filling temperature in determining rice grain quality (Chen et al., 2017; Tang et al., 2018), the present results suggest that the contribution of pre-anthesis climatic conditions has been underestimated. Notably, compared with indica inbred rice, super hybrid rice typically has larger panicles, indicating a greater sink size and a stronger need for assimilate accumulation before anthesis (Ohsumi et al., 2011). Moreover, differential grain yield responses of hybrid and inbred rice to minimum temperature have also been reported, suggesting that hybrid rice is more sensitive to pre-anthesis climatic variation (Huang et al., 2021). These findings highlight the importance of considering pre-anthesis climatic conditions when assessing grain quality in super hybrid rice with large panicles. In particular, pre-anthesis solar radiation was positively associated with milling performance, probably through its effects on canopy photosynthesis, dry matter accumulation, and assimilate supply before grain filling. The difference between 2021 and 2022 further showed the effect of climatic variation on grain quality. At Longhui, the post-anthesis daily maximum temperature was higher in 2022 than in 2021 (33.74 and 32.88 °C), and the daily temperature range also increased from 10.71 °C in 2021 to 13.54 °C in 2022. In addition, post-anthesis daily solar radiation increased from 17.98 MJ/m^2^ in 2021 to 22.81 MJ/m^2^ in 2022. These differences may partly explain the stronger variation of some appearance traits, especially chalkiness. For example, although YLY900 generally maintained the lowest chalkiness among the three varieties, its chalkiness at Longhui in 2022 was higher than that in 2021 under nitrogen application, suggesting that the appearance quality of modern super hybrid rice can still be affected by year-specific climatic conditions. High temperature before anthesis may influence photosynthetic capacity and assimilate accumulation, whereas high temperature after anthesis directly affects starch biosynthesis and endosperm filling. Therefore, production strategies for super hybrid rice should consider both pre-anthesis and post-anthesis temperature conditions, especially under years with extreme high temperature.

In this study, nitrogen application showed trait-specific effects on grain quality. It consistently increased protein content and also contributed to changes in milling and appearance traits under some treatment combinations. These results agree with previous reports that nitrogen mainly affects protein accumulation (Chen et al., 2012; Li et al., 2023). The relatively limited effects of nitrogen on other grain quality traits likely reflect the strong dependence of these traits on nitrogen application rate and timing (Wei et al., 2018). For example, moderate nitrogen application can reduce chalkiness, whereas excessive nitrogen tends to increase chalky grains and deteriorate appearance quality, as observed in both indica rice (Zhou et al., 2015) and super hybrid rice (Yang et al., 2025b). Consistent with earlier studies, reducing total nitrogen by 15–20% did not substantially affect grain quality in super hybrid such as LYPJ and YLY1 (Huang et al., 2017b). These results suggest that excessive nitrogen is not necessary to maintain grain quality in super hybrid rice. From a sustainability perspective, optimized nitrogen management is also important for reducing environmental risks in paddy soils, because different nitrogen management strategies can influence pollutant mobilization and greenhouse gas emissions in flooded rice systems (Wang et al., 2024). In addition, advances in biological nitrogen fixation and ecosystem-scale nitrogen management provide broader directions for reducing dependence on excessive synthetic N input in future agricultural systems (Cheng et al., 2026; Zhi et al., 2026). Although high nitrogen is still common in commercial production, especially for high-yielding varieties with large panicles (Wang and Peng, 2017), optimizing nitrogen management to balance yield, quality and environmental sustainability is essential for sustainable production. Future climate change may further modify the interactions among variety identity, nitrogen management, and climatic conditions. Therefore, cultivar-specific nitrogen management and climate-adaptive cultivation practices should be developed together for super hybrid rice production.

## 5 Conclusions

This study integrates historical breeding data with multi-site field experiments to examine long-term changes and environmental influences on grain quality in super hybrid rice. super hybrid rice varieties showed clear improvements in key quality traits, including higher head rice rate and lower chalkiness and amylose content.

Variety was the main driver of variation in milling quality, appearance traits, and amylose content, whereas nitrogen showed trait-specific effects, especially on protein content. Climatic factors, including both pre-anthesis and post-anthesis phases, consistently ranked among the top contributors to grain quality variation and influenced a broader range of traits than nitrogen. Overall, these findings show that past breeding has substantially improved the grain quality of super hybrid rice. Future improvements should continue to prioritize genetic enhancement, especially for higher head rice rate, lower chalkiness and suitable amylose content. Nitrogen management should avoid unnecessary increases in protein content while maintaining milling quality. Climate-adaptive practices should also be considered for both pre-anthesis and post-anthesis periods, including improving canopy growth before anthesis and reducing high-temperature exposure during grain filling.

## Data Availability

Data will be made available on request. Requests to access the datasets should be directed to Wanju Shi, swanju@126.com.

## References

[B1] Buggenhout J. Brijs K. Celus I. Delcour J. A. (2013). The breakage susceptibility of raw and parboiled rice: A review. J. Food Eng 117, 304–315. doi: 10.1016/j.jfoodeng.2013.03.00942574925

[B2] Chen J. Tang L. Shi P. Yang B. Sun T. Cao W. . (2017). Effects of short-term high temperature on grain quality and starch granules of rice (*Oryza sativa* L.) at post-anthesis stage. Protoplasma. 254, 935–943. doi: 10.1007/s00709-016-1002-y 27447698

[B3] Chen Y. Wang M. Ouwerkerk P. B. F. (2012). Molecular and environmental factors determining grain quality in rice. Food Energy Secur 1, 111–132. doi: 10.1002/fes3.1142587375

[B4] Cheng S. Cao L. Zhuang J. Chen S. Zhan X. Fan Y. . (2007). Super hybrid rice breeding in China: Achievements and prospects. J. Integr. Plant Biol. 49, 805–810. doi: 10.1111/j.1744-7909.2007.00514.x40046247

[B5] Cheng Q. Ma C. Wang X. (2026). Biological nitrogen and carbon fixation: Bridging the gap between synthetic symbioses and synthetic biology. Crop J. 14, 74–86. doi: 10.1016/j.cj.2026.01.00242574925

[B6] Custodio M. C. Cuevas R. P. Ynion J. Laborte A. G. Velasco M. L. Demont M. (2019). Rice quality: How is it defined by consumers, industry, food scientists, and geneticists? Trends Food Sci. Technol. 92, 122–137. doi: 10.1016/j.tifs.2019.07.039 31787805 PMC6876681

[B7] Deng J. Ye J. Liu K. Harrison M. T. Zhong X. Wang C. . (2022). Optimizing agronomy improves super hybrid rice yield and nitrogen use efficiency through enhanced post-heading carbon and nitrogen metabolism. Agronomy. 13, 13. doi: 10.3390/agronomy1301001330654563

[B8] Feng F. Li Y. Qin X. Liao Y. Siddique K. H. M. (2017). Changes in rice grain quality of indica and japonica type varieties released in China from 2000 to 2014. Front. Plant Sci. 8, 1863. doi: 10.3389/fpls.2017.01863 29163589 PMC5671604

[B9] Fitzgerald M. A. McCouch S. R. Hall R. D. (2009). Not just a grain of rice: The quest for quality. Trends Plant Sci. 14, 133–139. doi: 10.1016/j.tplants.2008.12.004 19230745

[B10] He H. Shi J. Chen M. Zhao J. Qu K. Li M. . (2025). Dynamic changes of rice sheath non-structural carbohydrates and source–sink balance under elevated atmospheric CO_2_ concentration and temperature stresses. J. Plant Physiol. 311, 154547. doi: 10.1016/j.jplph.2025.154547 40527110

[B11] Huang M. Shan S. Chen J. Cao F. Jiang L. Zou Y. (2017b). “ Comparison on grain quality between super hybrid and popular inbred rice cultivars under two nitrogen management practices,” in Advances in International Rice Research. Ed. Li J. ( IntechOpen, London), 111. doi: 10.5772/66386

[B12] Huang L. Sun F. Yuan S. Peng S. Wang F. (2018). Different mechanisms underlying the yield advantage of ordinary hybrid and super hybrid rice over inbred rice under low and moderate N input conditions. Field Crops Res. 216, 150–157. doi: 10.1016/j.fcr.2017.11.01942574925

[B13] Huang M. Tang Q. Ao H. Zou Y. (2017a). Yield potential and stability in super hybrid rice and its production strategies. J. Integr. Agric. 16, 1009–1017. doi: 10.1016/S2095-3119(16)61535-6

[B14] Huang L. Wang F. Liu Y. Zhang Y. (2021). Night temperature determines the interannual yield variation in hybrid and inbred rice widely used in central China through different effects on reproductive growth. Front. Plant Sci. 12, 646168. doi: 10.3389/fpls.2021.646168 34149750 PMC8212977

[B15] Li R. Li M. Ashraf U. Liu S. Zhang J. (2019). Exploring the relationships between yield and yield-related traits for rice varieties released in China from 1978 to 2017. Front. Plant Sci. 10, 543. doi: 10.3389/fpls.2019.00543 31134107 PMC6514245

[B16] Li L. Shi S. Cheng B. Zhao D. Pan K. Cao C. . (2023). Association between rice protein components and eating quality traits of different rice varieties under different nitrogen levels. J. Cereal Sci. 113, 103760. doi: 10.1016/j.jcs.2023.10376042574925

[B17] Li J. Zhang Y. Wu J. Shu Y. Zhou P. Deng Q. (2014). Cultivation practices of super high-yielding combination Yliangyou900 with a maximum grain yield of 15.40 t ha^–1^. China Rice 20, 1–4. doi: 10.3969/j.issn.1006-8082.2014.06.001

[B18] Lin C. Li C. Lin S. Yang F. Huang J. Liu Y. . (2010). Influence of high temperature during grain filling on the accumulation of storage proteins and grain quality in rice (*Oryza sativa* L.). J. Agric. Food. Chem. 58, 10545–10552. doi: 10.1021/jf101575j 20839801

[B19] Lin X. Zhu D. Chen H. Zhang Y. (2009). Effects of plant density and nitrogen application rate on grain yield and nitrogen uptake of super hybrid rice. Rice Sci. 16, 138–142. doi: 10.1016/S1672-6308(08)60070-0

[B20] Liu K. Deng J. Lu J. Wang X. Lu B. Tian X. . (2019). High nitrogen levels alleviate yield loss of super hybrid rice caused by high temperatures during the flowering stage. Front. Plant Sci. 10, 357. doi: 10.3389/fpls.2019.00357 30972091 PMC6443885

[B21] Lu Y. Tang Y. Zhang J. Liu S. Liang X. Li M. . (2024). Variations and trends in rice quality across different types of approved varieties in China 1978–2022. Agronomy. 14, 1234. doi: 10.3390/agronomy1406123430654563

[B22] Ohsumi A. Takai T. Ida M. Yamamoto T. Arai-Sanoh Y. Yano M. . (2011). Evaluation of yield performance in rice near-isogenic lines with increased spikelet number. Field Crops Res. 120, 68–75. doi: 10.1016/j.fcr.2010.08.01342574925

[B23] Qian Q. Guo L. Smith S. M. Li J. (2016). Breeding high-yield superior quality hybrid super rice by rational design. Natl. Sci. Rev. 3, 283–294. doi: 10.1093/nsr/nww006

[B24] Sen S. Chakraborty R. Kalita P. (2020). Rice—not just a staple food: A comprehensive review on its phytochemicals and therapeutic potential. Trends Food Sci. Technol. 97, 265–285. doi: 10.1016/j.tifs.2020.01.02242574925

[B25] Talla A. Swain D. K. Srivastava R. K. Tewari V. K. Purohit S. (2025). Integrating climatic factors and agronomic management for optimal hybrid rice yield and quality. Commun. Soil Sci. Plant Anal. 56, 1044–1059. doi: 10.1080/00103624.2024.244007137339054

[B26] Tang S. Chen W. Liu W. Zhou Q. Zhang H. Wang S. . (2018). Open-field warming regulates the morphological structure, protein synthesis of grain and affects the appearance quality of rice. J. Cereal Sci. 84, 20–29. doi: 10.1016/j.jcs.2018.09.01342574925

[B27] Tang L. Wu A. Li S. Tuerdimaimaiti M. Zhang G. (2023). Impacts of climate change on rice grain: A literature review on what is happening, and how should we proceed? Foods. 12, 536. doi: 10.3390/foods12030536 36766065 PMC9914188

[B28] Tang S. Zhang H. Liu W. Dou Z. Zhou Q. Chen W. . (2019). Nitrogen fertilizer at heading stage effectively compensates for the deterioration of rice quality by affecting the starch-related properties under elevated temperatures. Food Chem. 277, 455–462. doi: 10.1016/j.foodchem.2018.10.137 30502170

[B29] Tu D. Yang L. Liang S. Li Z. Xu Y. Zhou Y. . (2025). Nitrogen management regulates grain quality of rice ratoon crop by altering starch structure and protein composition. Carbohydr Polym 374, 124721. doi: 10.1016/j.carbpol.2025.124721 41371860

[B30] Wang Q. Huang J. He F. Cui K. Zeng J. Nie L. . (2012). Head rice yield of “super” hybrid rice Liangyoupeijiu grown under different nitrogen rates. Field Crops Res. 134, 71–79. doi: 10.1016/j.fcr.2012.05.00142574925

[B31] Wang F. Peng S. (2017). Yield potential and nitrogen use efficiency of China’s super rice. J. Integr. Agric. 16, 1000–1008. doi: 10.1016/S2095-3119(16)61561-7

[B32] Wang F. Zhang J. Zeng Y. Wang H. Zhao X. Chen Y. . (2024). Arsenic mobilization and nitrous oxide emission modulation by different nitrogen management strategies in a flooded ammonia-enriched paddy soil. Pedosphere 34, 1051–1065. doi: 10.1016/j.pedsph.2023.09.00842574925

[B33] Wei H. Zhu Y. Qiu S. Han C. Hu L. Xu D. . (2018). Combined effect of shading time and nitrogen level on grain filling and grain quality in japonica super rice. J. Integr. Agric. 17, 2405–2417. doi: 10.1016/S2095-3119(18)62025-8

[B34] Xiong R. Tan X. Yang T. Wang H. Pan X. Zeng Y. . (2023). Starch multiscale structure and physicochemical property alterations in high-quality indica rice and cooked rice texture under different nitrogen panicle fertilizer applications. Int. J. Biol. Macromol 252, 126455. doi: 10.1016/j.ijbiomac.2023.126455 37633549

[B35] Xu Y. Guan X. Han Z. Zhou L. Zhang Y. Asad M. A. . (2022). Combined effect of nitrogen fertilizer application and high temperature on grain quality properties of cooked rice. Front. Plant Sci. 13, 874033. doi: 10.3389/fpls.2022.874033 35519803 PMC9062220

[B36] Xu L. Yuan S. Wang X. Yu X. Peng S. (2021). High yields of hybrid rice do not require more nitrogen fertilizer than inbred rice: A meta-analysis. Food Energy Secur 10, 341–350. doi: 10.1002/fes3.27642587375

[B37] Yang X. Liu L. Mahmood N. Qin J. Zhao Q. Song X. (2025b). Response of texture, rheological characteristics and starch molecular structure of super hybrid indica rice to different nitrogen fertilizer levels. Carbohydr Polym 366, 123927. doi: 10.1016/j.carbpol.2025.123927 40733844

[B38] Yang H. Wu L. Zhang B. Yang T. Zou J. (2025a). Temporal changes in grain yield and quality of rice varieties released in Guangdong Province, China, (1990–2020). Front. Agron. 7, 1604840. doi: 10.3389/fagro.2025.1604840

[B39] Yuan L. (2015). Development of super hybrid rice for food security in China. Engineering. 1, 13–14. doi: 10.15302/J-ENG-2015021

[B40] Zeng Y. Tan X. Zeng Y. Xie X. Pan X. Shi Q. . (2019). Changes in the rice grain quality of different high-quality rice varieties released in southern China from 2007 to 2017. J. Cereal Sci. 87, 111–116. doi: 10.1016/j.jcs.2019.03.01542574925

[B41] Zhang H. Xu H. Jiang Y. Zhang H. Wang S. Wang F. . (2021). Genetic control and high temperature effects on starch biosynthesis and grain quality in rice. Front. Plant Sci. 12, 757997. doi: 10.3389/fpls.2021.757997 34975940 PMC8718882

[B42] Zhang X. Zhang Q. Yang J. Jin Y. Wu J. Xu H. . (2023). Comparative effects of heat stress at booting and grain-filling stage on yield and grain quality of high-quality hybrid rice. Foods. 12, 4093. doi: 10.3390/foods12224093 38002151 PMC10670581

[B43] Zhen F. Zhou J. Mahmood A. Wang W. Chang X. Liu B. . (2020). Quantifying the effects of short-term heat stress at booting stage on nonstructural carbohydrates remobilization in rice. Crop J. 8, 194–212. doi: 10.1016/j.cj.2019.07.002 38592815 PMC10975544

[B44] Zhi L. Li X. Bai J. Wang D. Yang J. Ren J. (2026). Unveiling critical nitrogen metabolism nodes and stakeholder priorities across coastal wetland eco-socioeconomic systems via integrated network analytics. Environ. Impact Assess Rev. 121, 108503. doi: 10.1016/j.eiar.2026.10850342574925

[B45] Zhou X. Chen D. Guo J. Chen P. Li L. Chen K. . (2022b). Genetic improvement of grain quality traits in indica inbred rice cultivars developed in South China during 1956–2020. Euphytica. 218, 8. doi: 10.1007/s10681-021-02953-230311153

[B46] Zhou T. Li Z. Li E. Wang W. Yuan L. Zhang H. . (2022a). Optimization of nitrogen fertilization improves rice quality by affecting the structure and physicochemical properties of starch at high yield levels. J. Integr. Agric. 21, 1576–1592. doi: 10.1016/S2095-3119(21)63678-X

[B47] Zhou L. Liang S. Ponce K. Marundon S. Ye G. Zhao X. (2015). Factors affecting head rice yield and chalkiness in indica rice. Field Crops Res. 172, 1–10. doi: 10.1016/j.fcr.2014.12.00442574925

[B48] Zhou H. Xia D. He Y. (2020). Rice grain quality—traditional traits for high quality rice and health-plus substances. Mol. Breed. 40, 1. doi: 10.1007/s11032-019-1080-630311153

