# Supplementary material for: Characterizing the long-term variation in grain quality of super hybrid rice under the interactive effects of nitrogen and climate
Source: Front Plant Sci. 2026 Sep 16;17:1820987. doi: 10.3389/fpls.2026.1820987 (PMC13623617; doi:10.3389/fpls.2026.1820987)
Supplement: Supplementary file 1 [file Table1.docx]

Supplementary Material

**Supplementary Table 1.** Information about the varieties used in the experiments.

| **Variety** | **Abbreviation** | **Female parent** | **Male parent** | **Release year** | **Subspecies** | **Grain Type** | **Maturity** |
| --- | --- | --- | --- | --- | --- | --- | --- |
| Liangyoupeijiu | LYPJ | Peiai64S | 9311 | 1999 | Indica | Long-grain | Mid-season |
| Y-Liangyou 1 | YLY1 | Y58S | 9311 | 2006 | Indica | Long-grain | Mid-season |
| Y-Liangyou 900 | YLY900 | Y58S | R900 | 2015 | Indica | Long-grain | Mid-season |

Note: The information was from the website of China Rice Data Center（<http://www.ricedata.cn/variety>）

**Supplementary Table 2.** Summary of analysis of variance for grain quality traits of super hybrid rice under site, variety, and nitrogen treatment in 2021 and 2022.

| **Trait** | **Brown rice rate** | |  | **Milled rice rate** | |  | **Head rice rate** | |  | **Grain length** | |  | **Grain width** | |  | **Grain aspect ratio** | |  | **Chalky grain rate** | |  | **Chalkiness degree** | |  | **Amylose content** | |  | **Protein content** | |
| --- | --- | --- | --- | --- | --- | --- | --- | --- | --- | --- | --- | --- | --- | --- | --- | --- | --- | --- | --- | --- | --- | --- | --- | --- | --- | --- | --- | --- | --- |
| Year | 2021 | 2022 |  | 2021 | 2022 |  | 2021 | 2022 |  | 2021 | 2022 |  | 2021 | 2022 |  | 2021 | 2022 |  | 2021 | 2022 |  | 2021 | 2022 |  | 2021 | 2022 |  | 2021 | 2022 |
| Site (S) | *** | *** |  | * | *** |  | *** | *** |  | *** | ** |  | *** | *** |  | *** | *** |  | *** | *** |  | *** | *** |  | *** | *** |  | *** | *** |
| Variety (V) | *** | *** |  | *** | *** |  | *** | *** |  | *** | *** |  | *** | *** |  | *** | *** |  | *** | *** |  | *** | *** |  | *** | *** |  | *** | *** |
| Nitrogen (N) | *** | *** |  | *** | *** |  | *** | *** |  | *** | *** |  | *** | *** |  | *** | *** |  | *** | *** |  | *** | *** |  | *** | *** |  | *** | *** |
| S × V | ** | ** |  | *** | *** |  | *** | *** |  | *** | *** |  | *** | * |  | *** | *** |  | *** | *** |  | *** | *** |  | *** | ** |  | *** | ns |
| S × N | ** | * |  | *** | *** |  | *** | *** |  | *** | *** |  | *** | *** |  | *** | *** |  | *** | *** |  | *** | *** |  | *** | * |  | *** | ns |
| V × N | * | ns |  | ** | *** |  | *** | *** |  | *** | *** |  | *** | ns |  | *** | *** |  | *** | *** |  | *** | *** |  | *** | *** |  | *** | * |
| S × V × N | *** | ns |  | *** | * |  | *** | *** |  | *** | *** |  | ** | ns |  | *** | * |  | *** | *** |  | *** | *** |  | *** | ** |  | ** | * |

Note: S, V, and N indicate site, variety, and nitrogen treatment, respectively. *, **, and *** indicate significance at *P < 0.05*, *P < 0.01*, and *P < 0.001*, respectively; ns indicates no significant difference.

**Supplementary Table 3.** Parameters and out-of-bag validation results of random forest models for grain quality traits.

| **Trait** | **n** | **nTrees** | **mtry** | **Min. node size** | **OOB MSE** | **OOB RMSE** | **OOB MAE** | **OOB mean relative error** | **OOB R²** |
| --- | --- | --- | --- | --- | --- | --- | --- | --- | --- |
| Brown rice | 48 | 300 | 3 | 8 | 15.23 | 3.90 | 3.38 | 0.0425 | 0.75 |
| Milled rice | 48 | 300 | 3 | 8 | 20.17 | 4.49 | 3.58 | 0.0505 | 0.75 |
| Head rice | 48 | 300 | 3 | 8 | 30.42 | 5.52 | 4.38 | 0.0905 | 0.70 |
| Grain length | 48 | 300 | 3 | 8 | 0.118 | 0.344 | 0.282 | 0.0442 | 0.76 |
| Grain width | 48 | 300 | 3 | 8 | 0.013 | 0.111 | 0.088 | 0.0413 | 0.76 |
| Grain aspect ratio | 48 | 300 | 3 | 8 | 0.031 | 0.175 | 0.141 | 0.0461 | 0.75 |
| Chalky grain rate | 48 | 300 | 3 | 8 | 40.35 | 6.35 | 4.96 | 0.1845 | 0.73 |
| Chalkiness degree | 48 | 300 | 3 | 8 | 5.48 | 2.34 | 1.88 | 0.1810 | 0.73 |
| Amylose content | 48 | 300 | 3 | 8 | 3.24 | 1.80 | 1.43 | 0.0802 | 0.79 |
| Protein content | 48 | 300 | 3 | 8 | 1.02 | 1.01 | 0.81 | 0.0909 | 0.80 |

Note: Treatment means across three replications were used for RF analysis. n = 48 for each trait. nTrees indicates the number of regression trees; mtry indicates the number of variables randomly selected at each split; OOB indicates out-of-bag; MSE, RMSE and MAE indicate mean squared error, root mean squared error and mean absolute error, respectively.
